# The impact of novel hormonal agents on fracture risk in prostate cancer patients: a nationwide population-based cohort study

**DOI:** 10.1038/s41598-024-73598-z

**Published:** 2024-11-04

**Authors:** Chia-Yen Lin, Chun-Li Wang, Cheng-Kuang Yang, Jian-Ri Li, Chuan-Shu Chen, Kun-Yuan Chiu, Ching-Heng Lin, Shian-Shiang Wang

**Affiliations:** 1 Department of Urology, Taichung Veterans General Hospital, 1650 Taiwan Boulevard Sect. 4, Taichung, 407219 Taiwan, ROC; 2 School of Medicine, Chung Shan Medical University, Taichung, Taiwan, ROC; 3 School of Medicine, National Yang Ming Chiao Tung University, Taipei, Taiwan, ROC; 4 Institute of Medicine, Chung Shan Medical University, Taichung, Taiwan, ROC; 5 Department of Family Medicine, Taichung Veterans General Hospital, Taichung, Taiwan, ROC; 6 Department of Medicine and Nursing, Hungkuang University, Taichung, Taiwan, ROC; 7 Department of Applied Chemistry, National Chi Nan University, Nantou, Taiwan, ROC; 8 Department of Medical Research, Taichung Veterans General Hospital, Taichung, Taiwan, ROC

**Keywords:** Cancer, Physiology, Diseases

## Abstract

Prostate cancer (PC) treatment, particularly androgen deprivation therapy (ADT), remains pivotal, albeit linked to increased fracture risk due to osteoporosis. The advent of novel hormonal agents (NHAs) has spurred inquiries into their influence on bone health. This study aimed to evaluate the impact of NHAs on bone health in patients receiving combination therapy. We conducted a retrospective analysis using Taiwan’s National Health Insurance Research Database, encompassing men aged 45 and above diagnosed with PC without bone metastasis and undergoing ADT between 2000 and 2018. The study involved 25,949 patients, categorized into those receiving standard ADT (*n* = 25,166) and those on NHA combination therapy (*n* = 783). Our analysis delved into fracture risk, comorbidities, and osteoporosis treatments. Patients on NHA combination therapy faced significantly higher risks of any osteoporotic fracture and major osteoporotic fracture than those on ADT alone (HR = 1.29, 95% CI 1.04–1.61; HR = 1.37, 95% CI 1.06–1.75, respectively). Notably, age emerged as a critical factor, with the highest risk observed in those aged 90 or above. The 5-year overall survival rates were lower for patients who experienced any osteoporotic fracture, major osteoporotic fracture, and hospitalization due to osteoporotic fracture compared to those who did not experience these fractures (51.5% vs. 56.5%, 47.1% vs. 56.7%, and 48.2% vs. 56.3%, respectively, *p* < 0.001). Furthermore, patients not using any bone-modifying agents had the highest risk for all fracture types. In conclusion, NHA combination therapy in PC patients potentially escalates the risk of osteoporotic fractures, especially in older individuals. Our findings underscore the pivotal role of osteoporosis treatments in preventing fractures, emphasizing the importance of evaluating fracture risk in patients undergoing NHA combination therapy.

**Supplementary Information:**

The online version contains supplementary material available at 10.1038/s41598-024-73598-z.

## Introduction

Prostate cancer (PC) accounts for 26% of all newly diagnosed cancers in men, projecting an estimated 248,530 new cases in the US in 2021^1^. Androgen deprivation therapy (ADT) remains the cornerstone treatment for high-risk localized, locally advanced or metastatic PC^2–4^. Clinical guidelines recommend continuing ADT in metastatic PC patients until the end of life, even beyond the castration-resistant phase^5^. Consequently, ADT exposure can extend across multiple years, regardless of whether it’s employed for locally advanced or metastatic disease. However, ADT can accelerate osteoclastic bone resorption, leading to diminished bone mineral density (BMD) and an elevated susceptibility to fractures^6,7^.

A study analyzing data from the Surveillance, Epidemiology, and End Results (SEER)-linked Medicare database revealed that 19.4% of patients who underwent ADT experienced fractures within 12 to 60 months after diagnosis, compared to only 12.6% of patients who did not receive ADT^8^. Later reports confirmed a consistent rise in fracture rates, with a 2-fold higher mortality among affected men^9^. Our previous study, based on the Taiwan National Health Insurance Research Database (NHIRD), also showed a 1.55 and 1.95 times higher risk of fractures associated with medical and surgical castration, respectively, in advanced prostate cancer patients compared with a non-cancer matched control group^10^. These findings align with another study that further corroborates the concept of an augmented fracture risk associated with ADT^11–13^.

Prior studies have established a noteworthy correlation between the number of gonadotropin-releasing hormone doses administered within the first year following diagnosis and the subsequent fracture risk^8^, underscoring the potential influence of hormonal suppression intensity on subsequent fracture susceptibility. Moreover, in the era of novel hormonal agents (NHA), combination therapy with conventional ADT has become the standard of care for metastatic PC. However, it is imperative to recognize that although NHA combination therapy can yield improved outcomes for PC patients, it may also heighten the vulnerability to osteoporotic fractures. In clinical trials involving NHA combination therapy for patients with non-metastatic castration-resistant prostate cancer (nmCRPC), both apalutamide and enzalutamide were linked to greater fracture risks in comparison to the placebo group (11.7% vs. 6.5% and 9.8% vs. 4.9%, respectively)^14,15^. A recent meta-analysis of randomized controlled trials (RCTs) in men with advanced and metastatic prostate cancer (PCa) undergoing novel hormonal agent (NHA) combination therapy revealed a significantly increased risk of fractures and falls^16^. Additionally, current guidelines on assessing and managing treatment-induced bone loss in prostate cancer patients highlight the need for further research into the impact of NHA combination therapy on bone health^17^.

Nonetheless, fractures as adverse events of ADT are multifactorial results, not only related to osteoporosis but also encompassing falls and mental disturbances. Previous epidemiological studies have shown the potential role of ADT’s psychological impacts in augmenting fall-related risks. Notably, men undergoing ADT for PC exhibited an elevated susceptibility to dementia and/or Alzheimer’s disease, consequently heightening the propensity for falls. This phenomenon was evident across studies conducted in both eastern and western populations, underscoring their collective contribution to elevated fracture rates, resulting in increased mortality and morbidity^18–21^. Moreover, the increased fracture risk among PC patients can also be attributed to several additional factors, including age, disease advancement, the employment of bone-modifying agents, and comorbidities^22,23^.

In this study, we utilized the Taiwan National Health Insurance Research Database (NHIRD) to evaluate the risk of osteoporotic fractures in men with PC who underwent NHA combination therapy without bone involvement. Additionally, we investigated whether osteoporosis treatment agents could mitigate any potential adverse effects.

## Results

### Demographic characteristics of study population

A total of 25,949 newly diagnosed patients with PC (ICD9-CM code: 185/ ICD10-CM code: C61) holding a certificate of catastrophic illness between 2000 and 2019 met the primary inclusion criteria. The definition of catastrophic illness is determined by the regulations of the Ministry of Health and Welfare, Taiwan^24,25^. These patients were divided into two distinct groups: those receiving ADT alone (*n* = 25,166) and those with a combination of conventional ADT and NHA (*n* = 783). Notably, there were differences between the cohorts concerning age, comorbidities, fracture status, and osteonecrosis of the jaw during bone antiresorptive treatment, as outlined in Table 1.

**Table 1:** Characteristics of patient with prostate cancer under androgen deprivation therapy(ADT) alone or novel hormonal agents(NHA) combination therapy.

Characteristic	Total	Conventional ADT group	NHA combination group	*P*-value
	*n* = 25,949	*n* = 25,166	*n* = 783	
	*n*	*n* (%)	*n* (%)	
Age				0.005
< 60	1230	1199 (4.8)	31 (4)	
60–69	5752	5556 (22.1)	196 (25)	
70–79	11,546	11,230 (44.6)	316 (40.4)	
80–89	6817	6607 (26.3)	210 (26.8)	
≥ 90	604	574 (2.3)	30 (3.8)	
Comorbidity				
Myocardial infarction	1030	1001 (4)	29 (3.7)	0.699
Heart failure	2775	2697 (10.7)	78 (10)	0.501
Peripheral vascular disease	1126	1090 (4.3)	36 (4.6)	0.719
Cerebrovascular Disease	6796	6615 (26.3)	181 (23.1)	0.047
Dementia	1427	1391 (5.5)	36 (4.6)	0.261
Pulmonary disease	11,895	11,586 (46)	309 (39.5)	< 0.001
Connective tissue disorder	614	593 (2.4)	21 (2.7)	0.555
Chronic liver disease	5490	5325 (21.2)	165 (21.1)	0.953
Diabetes mellitus	9025	8675 (34.5)	350 (44.7)	< 0.001
Paraplegia	567	543 (2.2)	24 (3.1)	0.087
Chronic kidney disease	4938	4747 (18.9)	191 (24.4)	< 0.001
Hyperthyroidism	234	228 (0.9)	6 (0.8)	0.684
Hyperparathyroidism	30	30 (0.1)	0 (0)	0.334
Steroid				< 0.001
No	8227	8146 (32.4)	81 (10.3)	
Yes	17,722	17,020 (67.6)	702 (89.7)	
Outcome				
Any osteoporotic fracture	3232	3148 (12.5)	84 (10.7)	0.137
Major osteoporotic fracture	2362	2298 (9.1)	64 (8.2)	0.359
Hospitalization related to osteoporotic fracture	1387	1355 (5.4)	32 (4.1)	0.112
Osteonecrosis of the jaw during bone antiresorptive treatment	46	26 (0.1)	20 (2.6)	< 0.001

In terms of age distribution, the majority of patients fell within the 70–79 age group, while the group receiving NHA combination therapy exhibited a higher proportion of patients in the 60–69 age range (25% vs. 22.1%) and a lower proportion in the 70–79 age range (40.4% vs. 44.6%) compared to the group receiving ADT alone. Additionally, there was a higher percentage of long-term steroid usage in the NHA combination group (89.7% vs. 67.7%).

**Fig. 1:**
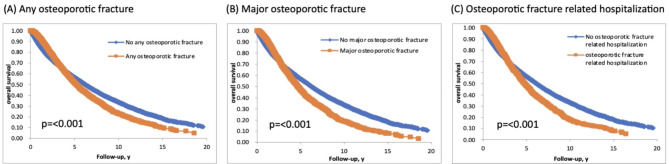
Overall survival curves of patients with or without any osteoporotic fracture (**A**), major osteoporotic fracture (**B**) and osteoporotic fracture related hospitalization (**C**) for 25,949 patients with prostate cancer undergoing androgen deprivation therapy.

A total of 3,232 cases of any osteoporotic fracture, 2,362 cases of major osteoporotic fracture, and 1,387 cases of hospitalization due to osteoporotic fracture were observed in the study. Furthermore, the 5-year overall survival rates of patients who experienced any osteoporotic fracture, major osteoporotic fracture, and hospitalization due to osteoporotic fracture were lower compared to those who did not experience these fractures (51.5% vs. 56.5%, 47.1% vs. 56.7%, and 48.2% vs. 56.3% respectively, *p* < 0.001) (Fig. 1).

In the group receiving conventional ADT alone, the frequency of all types of fracture-related outcomes was slightly higher than in the group receiving NHA combination therapy. Additionally, the group receiving NHA combination therapy exhibited a significantly higher incidence of osteonecrosis of the jaw during bone antiresorptive treatment.

### Association of NHA combination and osteoporotic fracture

**Fig. 2:**
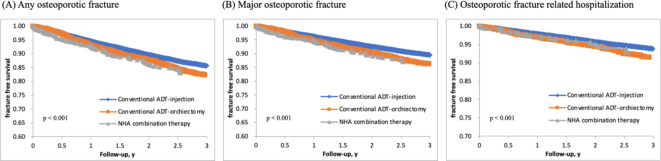
Survival curves for different types of fractures (any osteoporotic fracture (**A**), major osteoporotic fracture (**B**), and osteoporotic fracture-related hospitalization (**C**)) among 25,949 patients with prostate cancer, with or without novel hormonal agents combination therapy.

Figure 2 illustrates fracture-free survival following the initiation of hormonal therapy. We classified the patients in the fracture-free survival curve into three groups: those on medical ADT, those who received bilateral orchiectomy, and those on NHA combination therapy. Our analysis showed that patients receiving NHA combination therapy and those who underwent bilateral orchiectomy had a higher risk of fractures and fracture-related hospitalization compared to those on medical ADT. Notably, patients on NHA combination therapy exhibited an even greater risk of any osteoporotic fracture and major osteoporotic fracture compared to those who received surgical castration therapy. Subsequently, we conducted a multivariate regression analysis, which revealed that patients receiving NHA combination therapy had a significantly higher risk of any osteoporotic fracture and major osteoporotic fracture (HR = 1.29, *p* = 0.022; HR = 1.38, *p* = 0.012). Furthermore, the risk of fractures and fracture-related hospitalization increased with patient age, in comparison to those younger than 60 years old. Notably, patients aged 90 years or older exhibited the highest risk across all types of fractures (Table 2). Patients with cerebrovascular disease or pulmonary disease also had a higher risk of fractures and fracture-related hospitalization. However, no increased risk of fractures was found in patients with long-term steroid usage after analysis.

**Table 2:** Multivariable analysis of factors associated osteoporotic fracture in patient with prostate cancer under androgen deprivation therapy.

Characteristic	Any osteoporotic fracture				Major osteoporotic fracture				Hospitalization related to osteoporotic fracture			
	HR	95%CI		*P*-value	HR	95%CI		*P*-value	HR	95%CI		*P*-value
NHA combination therapy	1.29	1.04	1.61	**0.022**	1.38	1.07	1.77	**0.012**	1.14	0.80	1.62	0.464
Age												
< 60	1.00				1.00				1.00			
60–69	1.10	0.88	1.37	0.399	1.20	0.90	1.58	0.211	1.69	1.10	2.61	**0.017**
70–79	1.51	1.22	1.86	**0.000**	1.75	1.34	2.29	**< 0.001**	2.60	1.71	3.95	**< 0.001**
80–89	2.13	1.72	2.63	**< 0.001**	2.82	2.16	3.70	**< 0.001**	4.00	2.62	6.10	**< 0.001**
≥ 90	2.40	1.80	3.20	**< 0.001**	3.33	2.37	4.68	**< 0.001**	4.92	2.98	8.11	**< 0.001**
Comorbidity												
Myocardial infarction	1.07	0.90	1.27	0.426	1.05	0.86	1.29	0.620	0.86	0.65	1.14	0.295
Heart failure	1.06	0.95	1.18	0.341	1.07	0.94	1.21	0.305	1.14	0.97	1.33	0.123
Peripheral vascular disease	1.05	0.90	1.23	0.542	0.98	0.81	1.19	0.856	1.10	0.87	1.38	0.447
Cerebrovascular Disease	1.13	1.04	1.22	**0.004**	1.13	1.03	1.24	**0.011**	1.24	1.10	1.39	**0.001**
Dementia	1.07	0.93	1.24	0.327	1.06	0.90	1.25	0.455	1.07	0.87	1.32	0.533
Pulmonary disease	1.26	1.17	1.36	**< 0.001**	1.26	1.16	1.38	**< 0.001**	1.18	1.05	1.32	**0.004**
Connective tissue disorder	1.16	0.95	1.43	0.154	1.15	0.91	1.47	0.241	0.92	0.66	1.30	0.650
Chronic liver disease	0.98	0.90	1.06	0.564	0.96	0.87	1.06	0.412	0.90	0.79	1.03	0.121
Diabetes mellitus	1.07	1.00	1.15	0.070	0.97	0.89	1.06	0.543	1.04	0.93	1.16	0.503
Paraplegia	1.15	0.92	1.43	0.234	1.31	1.02	1.67	**0.036**	1.14	0.82	1.60	0.432
Chronic kidney disease	1.00	0.92	1.09	0.990	0.99	0.89	1.10	0.847	1.00	0.87	1.14	0.948
Hyperthyroidism	1.06	0.75	1.49	0.742	1.07	0.72	1.61	0.727	1.03	0.61	1.75	0.901
Hyperparathyroidism	0.53	0.13	2.13	0.373	0.39	0.05	2.74	0.342	1.34	0.33	5.37	0.680
Steroid												
No	1.00				1.00				1.00			
Yes	1.00	0.92	1.08	0.924	0.96	0.87	1.05	0.340	1.00	0.89	1.13	0.967

### Association of bone modifying agents and osteoporotic fractures

We conducted an analysis to evaluate the impact of bisphosphonates and denosumab in the prevention of fractures. Compared to patients who received denosumab, those who did not use any bone modifying agent had the highest risk of all types of fractures (any osteoporotic fracture, HR = 1.76, 95% CI 1.41 to 2.19, *p* < 0.001; major osteoporotic fracture, HR = 1.92, 95% CI 1.47 to 2.52, *p* < 0.001; hospitalizations related to osteoporotic fracture, HR = 2.11, 95% CI 1.47 to 3.01, *p* < 0.001).

Furthermore, patients who used bisphosphonates also had a higher risk of all types of fractures compared to those who received denosumab (any osteoporotic fracture, HR = 1.43, 95% CI 1.11 to 2.84, *p* = 0.006; major osteoporotic fracture, HR = 1.65, 95% CI 1.22 to 2.24, *p* = 0.001; hospitalizations related to osteoporotic fracture, HR = 1.70, 95% CI 1.14 to 2.53, *p* = 0.010). Interestingly, the risk of osteonecrosis related to medication was lower in the bisphosphonates group (HR = 0.13, 95% CI 0.06 to 0.26, *p* < 0.001) (Table 3). When considering the interaction between the osteoporotic effects of NHA and different bone-modifying agents, patients undergoing conventional ADT with denosumab prevention exhibited the lowest risk of all types of fractures, followed by patients undergoing NHA combination therapy with denosumab prevention.

**Table 3:** Impact of novel hormonal agents combination therapy and bone modifying agents on osteoporotic fractures.

Characteristic	Any osteoporotic fracture						Major osteoporotic fracture						
	No, *n*(%)	Yes, *n*(%)	HR	95%CI		*P*-value	No, *n*(%)	Yes, *n*(%)	HR	95%CI		*P*-value	
Bone modifying agents													
Denosumab	1153 (93.4)	81 (6.6)	1.00				1189 (95.7)	54 (4.3)	1.00				
BP	2669 (91.9)	236 (8.1)	1.43	1.11	1.84	**0.006**	2748 (93.8)	183 (6.2)	1.65	1.22	2.24	**0.001**	
No use	18,895 (86.6)	2915 (13.4)	1.76	1.41	2.19	**<0.001**	19,650 (90.2)	2125 (9.8)	1.92	1.47	2.52	**<0.001**	

Hormone therapy	Bone modifying agents	Any osteoporotic fracture						Major osteoporotic fracture					
		No, n(%)	Yes, n(%)	HR	95%CI		P-value	No, n(%)	Yes, n(%)	HR	95%CI		P-value
ADT alone	Denosumab	858 (93.3)	62 (6.7)	1.00				889 (95.7)	40 (4.3)	1.00			
	BP	2555 (92.0)	223 (8.0)	1.50	1.13	1.99	**0.005**	2628 (93.7)	176 (6.3)	1.83	1.30	2.59	**0.001**
	No use	18,605 (86.7)	2863 (13.3)	1.88	1.46	2.41	**<0.001**	19,351 (90.3)	2082 (9.7)	2.13	1.56	2.91	**<0.001**
NHA combination	Denosumab	295 (94.0)	19 (6.1)	1.42	0.85	2.37	0.182	300 (95.5)	14 (4.5)	1.68	0.92	3.09	0.094
	BP	114 (89.8)	13 (10.2)	2.49	1.37	4.52	**0.003**	120 (94.5)	7 (5.5)	2.10	0.94	4.70	0.070
	No use	290 (84.8)	52 (15.2)	3.00	2.07	4.34	**<0.001**	299 (87.4)	43(12.6)	3.87	2.51	5.95	**<0.001**

Characteristic	Hospitalization related to osteoporotic fracture						Osteonecrosis due to drugs						
	No, *n*(%)	Yes, *n*(%)	HR	95%CI		*P*-value	No, *n*(%)	Yes, *n*(%)	HR	95%CI		*P*-value	
Bone modifying agents													
Denosumab	1283 (97.6)	31 (2.4)	1.00				1344 (97.3)	37 (2.7)	1.00				
BP	2952 (96.5)	107 (3.5)	1.70	1.14	2.53	**0.010**	3158 (99.7)	9 (0.3)	0.14	0.07	0.28	**<0.001**	
No use	20,327 (94.2)	1249 (5.8)	2.11	1.47	3.01	**<0.001**	21,401 (100.0)	0 (0.0)	-				

Hormone therapy	Bone modifying agents	Hospitalization related to osteoporotic fracture						Osteonecrosis due to drugs					
		No, n(%)	Yes, n(%)	HR	95%CI		P-value	No, n(%)	Yes, n(%)	HR	95%CI		P-value
ADT alone	Denosumab	971 (97.6)	24 (2.4)	1.00				1035 (97.8)	23(2.2)	1.00			
	BP	2828 (96.5)	102 (3.5)	1.81	1.16	2.82	**0.009**	3034 (99.9)	3 (0.1)	0.06	0.02	0.19	**<0.0001**
	No use	20,012 (94.2)	1229 (5.8)	2.26	1.51	3.39	**<0.001**	21,071 (100.0)	0 (0.0)	-			
NHA combination	Denosumab	312 (97.8)	7 (2.2)	1.49	0.64	3.45	0.358	309 (95.7)	14 (4.3)	2.67	1.34	5.32	**0.005**
	BP	124 (96.1)	5 (3.9)	2.47	0.94	6.49	0.065	124 (95.4)	6 (4.6)	2.84	1.14	7.10	**0.025**
	No use	315 (94.0)	20 (6.0)	3.22	1.78	5.84	**<0.001**	330 (100.0)	0 (0.0)	-			

ADT, androgen deprivation therapy; BP, bisphosphonate; NHA, Novel hormonal agents.

Moreover, the clinical characteristics revealed that patients treated with denosumab had higher percentages of several fracture-predisposing factors compared to those treated with bisphosphonates. These factors include age over 80 years (31.4% vs. 26.5%), NHA combination therapy (17.1% vs. 3.3%, *p* < 0.001), cerebrovascular disease (27.2% vs. 22.7%, *p* < 0.001), pulmonary disease (47.3% vs. 43.2%, *p* = 0.003), and long-term steroid usage (76.9% vs. 62.6%, *p* < 0.001)(Table 4).

**Table 4:** Characteristics of patient with prostate cancer under different bone modifying agents.

Characteristic	Denosumab group	Bisphosphonate group	Total	*P*-value
	(*n*=1904)	(*n*=3974)		
	*n* (%)	*n* (%)	*n* (%)	
Age				**<0.001**
<60	112 (5.9)	254 (6.4)	366	
60-69	415 (21.8)	890 (22.4)	1305	
70-79	778 (40.9)	1779 (44.8)	2557	
80-89	541 (28.4)	992 (25)	1533	
≥90	58 (3)	59 (1.5)	117	
Hormone therapy				**<0.001**
ADT alone	1578 (82.9)	3844 (96.7)	5422	
NHA combination	326 (17.1)	130 (3.3)	456	
Comorbidity				
Myocardial infarction	71 (3.7)	117 (2.9)	188	0.110
Heart failure	194 (10.2)	434 (10.9)	628	0.395
Peripheral vascular disease	88 (4.6)	153 (3.9)	241	0.163
Cerebrovascular Disease	517 (27.2)	901 (22.7)	1418	**<0.001**
Dementia	106 (5.6)	160 (4)	266	**0.008**
Pulmonary disease	901 (47.3)	1718 (43.2)	2619	**0.003**
Connective tissue disorder	51 (2.7)	96 (2.4)	147	0.546
Chronic liver disease	433 (22.7)	715 (18)	1148	**<0.001**
Diabetes mellitus	738 (38.8)	1078 (27.1)	1816	**<0.001**
Paraplegia	45 (2.4)	67 (1.7)	112	0.075
Chronic kidney disease	410 (21.5)	568 (14.3)	978	**<0.001**
Hyperthyroidism	22 (1.2)	25 (0.6)	47	0.034
Hyperparathyroidism				0.829
Steroid				**<0.001**
No	439 (23.1)	1486 (37.4)	1925	
Yes	1465 (76.9)	2488 (62.6)	3953	

## Discussion

To our knowledge, this study is the first population-based analysis to examine the osteoporotic fracture risk in patients with PC without bone metastasis undergoing NHA combination therapy. Our findings indicate that patients under NHA combination therapy face a 1.29-fold and 1.37-fold increased risk of any and major fractures, respectively. This elevated risk might result from the negative impact of androgen suppression on bone health.

In our study, where over 70% of patients were equal to or older than 70 years old, the multivariate analysis indicated that age remains a crucial factor influencing fracture risk. Despite the majority of patients with PC being over 70 years old and experiencing age-related bone loss and an increased risk of falls^17^, osteoporosis remains one of the most overlooked comorbidities of PC. Additionally, androgen deprivation therapy, the primary treatment for locally advanced or metastatic PC, has been shown to negatively impact bone health^6^. Notably, a substantial prevalence of up to 80.6% of osteoporosis has been reported in hormone-naive men with PC after 10 years of receiving ADT^26^. Indeed, specific guidelines have been released for the management of bone fragility in patients with prostate cancer^27–29^. However, in retrospective analyses of real-world databases up to 2015, only 8.4–23.4% of patients underwent BMD screening^30,31^. Thus, osteoporosis remains a commonly disregarded aspect of prostate cancer management in real-world clinical practice.

A study analyzing data from the SEER-Medicare database between 1992 and 1997 found that 19.4% of patients developed a fracture between 12 and 60 months after initiated ADT, whereas only 12.6% of patients who did not receive ADT experienced fractures^8^. Furthermore, this study also indicated a dose-related risk of sequential fractures in conventional ADT, and our findings further support this concept.

Moreover, The increased risk of fracture may also be a complication resulting from the adverse effects of NHA. The PROSPER and SPARTAN trials of nmCRPC setting with enzalutamide and apalutamide revealed a higher risk of falls, cognitive impairment, and memory/mental impairment compared to the placebo group^14,15,32,33^. On the other hand, darolutamide in the ARAMIS trial demonstrated a similar risk to the placebo arm in these aspects^34,35^. A possible explanation for this difference lies in the drug’s permeability across the blood-brain barrier and its impact on cerebral blood flow (CBF). Enzalutamide and apalutamide, as androgen receptor (AR) inhibitors, have higher brain: plasma ratios of 27% and 62%, respectively, in mice, while darolutamide’s ratio is only 1.9–3.9%.^36–38^ Additionally, a phase I trial using MRI imaging further investigated the effect of CBF of darolutamide and enzalutamide. It showed a significant reduction in CBF with enzalutamide, while darolutamide did not significantly alter CBF, aligning with its low blood-brain barrier penetration^39^. Nowadays, the early and extended use of second-generation AR inhibitors is recommended in metastatic PC and CRPC. Fracture prevention in these populations should no longer be overlooked.

The bone mineral density (BMD) screening is the primary tool for diagnosing osteoporosis. Over the past decades, the significance of fracture prevention in men with PC and the recommendation for BMD screening in guidelines have garnered increasing attention among physicians. However, the actual rates of BMD screening remained below expectations. A study analyzing the SEER-Medicare database showed that the BMD screening rate in men with nonmetastatic PC under ADT increased only from 6.8% in 2005 to 8.4% in 2015^30^. Similarly, a study utilizing the Quebec public health care insurance database reported a modest rise in BMD testing rates from 4.1% in 2000 to 23.4% in 2015^31^. These rates are still suboptimal, especially considering the era of NHA combination therapy, which was found to carry a higher risk of osteoporotic fractures in our study and is now the mainstay treatment for metastatic PC.

## Limitations

Our study has several limitations to consider. Using claims data, we cannot rule out that some fractures may be due to metastatic processes instead of osteoporosis, despite our focus on patients without bone metastasis. Furthermore, NHA reimbursement in Taiwan is limited to high-risk and high-volume mCRPC or mHSPC patients, potentially introducing baseline differences between our patient groups. The inherent constraints of observational claims research also affect our analysis. Despite our efforts to control for confounding factors, some latent confounders may remain unaccounted for. Additionally, our use of Charlson scores to assess comorbidities may not capture all fracture-related risk factors.

## Conclusion

In this cohort study, we revealed that PC patients, without bone metastasis, undergoing NHA combination therapy face a heightened risk of osteoporotic fractures. Age significantly influences this risk, particularly in older patients. Osteoporosis, an underrated comorbidity, remains a pressing concern. However, bone-modifying agents, especially denosumab, offer hope for fracture prevention. These findings emphasize the need to recognize osteoporosis in prostate cancer management and to implement proactive strategies, including bone density screening and the use of bone-modifying agents, to prevent deterioration in patients’ quality of life due to fractures.

## Materials and methods

The study protocol (approved no. CE13151-1) has been approved by the institutional review board at Taichung Veterans General Hospital. As this study solely utilized deidentified data, the acquisition of informed consent has been waived. All methods were conducted in accordance with the Declaration of Helsinki, and relevant guidelines and regulations.

### Data sources

The NHIRD encompasses comprehensive clinical and in-hospital data, including disease profiles, medical costs, and diagnostic codes, covering over 99% of Taiwan’s population. Furthermore, the Taiwan Cancer Registry (TCR) files provide comprehensive laboratory values and detailed clinical information on patients, ensuring the accuracy of cancer registration data. If any discrepancies were identified, they would collaborate with hospitals to verify and rectify the information.

### Defining the target population

We conducted a retrospective study on a cohort of men aged 45 years and older who had a confirmed histological diagnosis of PC between January 1, 2000, and June 30, 2018. Data analysis was performed from May 1 to June 30, 2023, with data censored at the last enrollment in the NHIRD. The inclusion criteria encompassed patients who underwent conventional androgen deprivation therapy (ADT), either through bilateral orchiectomy or medical castration. These patients were then categorized into groups based on whether they received conventional ADT alone or in combination with novel hormonal agents (NHA). It’s important to note that the observation period was set for a minimum of 3 years to explore potential bone-related adverse effects. Worth mentioning is that Taiwan’s national health insurance began covering abiraterone payments in December 2014 and enzalutamide payments in September 2016. In this study design, we excluded patients previously diagnosed with bone metastasis, whether they had received an operation or not, to minimize the effect of pathologic fractures related to prostate cancer. Additionally, to clarify the osteoporotic effects of ADT and minimize underlying fragility issues, we excluded patients who experienced any fractures 12 months prior to or 6 months after the initiation of ADT. Patients who received any bone-modifying agent (BMA) were presumed to be due to concerns of osteoporosis rather than bone metastasis or previous fractures. Consequently, the prevalence rate of BMA use was relatively low, at only 16% (4319 out of 25,949 patients). All fracture episodes were recorded after 6 months of initiating ADT. An illustration of the study flow diagram, outlining the principles used for patient selection and sampling (Supplement Fig. 1).

### Outcome of interest

The primary outcomes of interest were fractures (any fracture, major osteoporotic fracture, and hospitalization due to osteoporotic fracture), as well as overall survival (OS). We analyzed the frequency of any fracture and major osteoporotic fracture (in the spine, upper arm, lower arm, hip, and other femur), investigating the impact of comorbidities (including age, myocardial infarction, congestive heart failure, peripheral vascular disease, cerebrovascular disease, dementia, chronic pulmonary disease, connective tissue disorder, chronic liver disease, diabetes mellitus, paraplegia, chronic kidney disease, hyperthyroidism, hyperparathyroidism and long-term steroid used, defined as regular prednisone usage of more than 5 mg per day for more than 3 months.) using the Charlson comorbidity index. Additionally, we examined the use of bone-modifying agents (bisphosphonates and denosumab) among a subset of participants and assessed their efficacy in reducing fracture risk. Fracture onset time was defined from the initial ADT claim to the first fracture, with censoring at the date of death or the last day of NHIRD coverage. Our data extended up to 2021, which was the last year included in our analysis. It’s essential to note that all fractures considered occurred after the initial ADT claim. Overall survival (OS) was measured from 6 months after the commencement of conventional ADT or NHA combination therapy to the date of death, with censoring at the last follow-up to ensure data accuracy.

### Statistical analysis

The demographic characteristics of this study were compared using the chi-square test for categorical variables and the t-test for continuous variables. The association between the prevalence of fracture among the divided groups was assessed using multivariate Cox proportional hazard regression to estimate the hazard ratio (HR) and its 95% confidence interval (CI). The statistical significance of the cumulative incidence curves was examined using a log-rank test, and the curves were plotted using the Kaplan-Meier method. All statistical analysis were conducted using SAS software version 9.2 (SAS Institute Inc., Cary, NC, USA). Statistical significance was determined with a p-value less than 0.05.

## Electronic supplementary material

Below is the link to the electronic supplementary material.

Supplementary Material 1

## Data Availability

The data presented in this study are available on request from the corresponding author.

## References

[1] Siegel, R. L., Miller, K. D., Fuchs, H. E. & Jemal, A. Cancer statistics. *CA Cancer J. Clin.* **71**(1): 7–33. (2021). 33433946 10.3322/caac.21654

[2] Parker, C., Gillessen, S., Heidenreich, A. & Horwich, A. Cancer of the prostate: ESMO clinical practice guidelines for diagnosis, treatment and follow-up. *Annals Oncology: Official J. Eur. Soc. Med. Oncol.* **26**(Suppl 5), v69–77 (2015). 10.1093/annonc/mdv222 26205393

[3] Bolla, M. et al. Long-term results with immediate androgen suppression and external irradiation in patients with locally advanced prostate cancer (an EORTC study): A phase III randomised trial. *Lancet* **360**(9327), 103–106 (2002). 12126818 10.1016/s0140-6736(02)09408-4

[4] Horwitz, E. M. et al. Ten-year follow-up of radiation therapy oncology group protocol 92 – 02: A phase III trial of the duration of elective androgen deprivation in locally advanced prostate cancer. *J. Clin. Oncol.* **26**(15), 2497–2504 (2008). 18413638 10.1200/JCO.2007.14.9021

[5] Saad, F. et al. 2022 Canadian Urological Association (CUA)-Canadian Uro Oncology Group (CUOG) guideline: Management of castration-resistant prostate cancer (CRPC). *Can. Urol. Assoc. J.* **16**(11), E506–e515 (2022). 36378572 10.5489/cuaj.8161 PMC9665314

[6] Bienz, M. & Saad, F. Androgen-deprivation therapy and bone loss in prostate cancer patients: A clinical review. *Bonekey Rep.* **4**, 716 (2015). 26131363 10.1038/bonekey.2015.85 PMC4478875

[7] Alibhai, S. M. H. et al. Bone health and bone-targeted therapies for nonmetastatic prostate Cancer: a systematic review and Meta-analysis. *Ann. Intern. Med.* **167**(5), 341–350 (2017). 28785760 10.7326/M16-2577

[8] Shahinian, V. B., Kuo, Y. F., Freeman, J. L. & Goodwin, J. S. Risk of fracture after androgen deprivation for prostate cancer. *N. Engl. J. Med.* **352**(2), 154–164 (2005). 15647578 10.1056/NEJMoa041943

[9] Beebe-Dimmer, J. L. et al. Timing of androgen deprivation therapy use and fracture risk among elderly men with prostate cancer in the United States. *Pharmacoepidemiol Drug Saf.* **21**(1), 70–78 (2012). 22114014 10.1002/pds.2258 PMC3313550

[10] Chen, W. C. et al. Conventional androgen deprivation therapy is associated with an increased risk of fracture in advanced prostate cancer, a nationwide population-based study. *PloS One*. **18**(1), e0279981 (2023). 36598910 10.1371/journal.pone.0279981 PMC9812325

[11] Wu, C. T., Yang, Y. H., Chen, P. C., Chen, M. F. & Chen, W. C. Androgen deprivation increases the risk of fracture in prostate cancer patients: A population-based study in Chinese patients. *Osteoporos. Int.* **26**(9), 2281–2290 (2015). 25990353 10.1007/s00198-015-3135-9

[12] Alibhai, S. M. et al. Fracture types and risk factors in men with prostate cancer on androgen deprivation therapy: A matched cohort study of 19,079 men. *J. Urol.* **184**(3), 918–923 (2010). 20643458 10.1016/j.juro.2010.04.068

[13] Lau, Y. K. et al. Fracture risk in androgen deprivation therapy: a Canadian population based analysis. *Can. J. Urol.* **16**(6), 4908–4914 (2009). 20003666

[14] Smith, M. R. et al. Apalutamide treatment and metastasis-free survival in prostate cancer. *N. Engl. J. Med.* **378**(15), 1408–1418 (2018). 29420164 10.1056/NEJMoa1715546

[15] Sternberg, C. N. et al. Enzalutamide and survival in nonmetastatic, castration-resistant prostate cancer. *N. Engl. J. Med.* **382**(23), 2197–2206 (2020). 32469184 10.1056/NEJMoa2003892

[16] Jones, C. et al. Risk of Fractures and falls in men with advanced or metastatic prostate cancer receiving androgen deprivation therapy and treated with novel androgen receptor signalling inhibitors: A systematic review and Meta-analysis of Randomised controlled trials. *Eur. Urol. Oncol.* (2024). 10.1016/j.euo.2024.01.016 38383277

[17] Brown, J. E. et al. Guidance for the assessment and management of prostate cancer treatment-induced bone loss. A consensus position statement from an expert group. *J. Bone Oncol.* **25**, 100311 (2020). 32995252 10.1016/j.jbo.2020.100311 PMC7516275

[18] Lonergan, P. E. et al. Androgen deprivation therapy and the risk of dementia after treatment for prostate cancer. *J. Urol.* **207**(4), 832–840 (2022). 34854749 10.1097/JU.0000000000002335

[19] Huang, W. K. et al. Type of androgen deprivation therapy and risk of dementia among patients with prostate cancer in Taiwan. *JAMA Netw. Open.* **3**(8), e2015189 (2020). 32865575 10.1001/jamanetworkopen.2020.15189 PMC7489824

[20] Zhang, W. et al. Review of gait, cognition, and fall risks with implications for fall prevention in older adults with dementia. *Dement. Geriatr. Cogn. Disord*. **48**(1–2), 17–29 (2019). 31743907 10.1159/000504340

[21] Buskbjerg, C. R. et al. Androgen deprivation therapy and cognitive decline-associations with brain connectomes, endocrine status, and risk genotypes. *Prostate Cancer Prostatic Dis.* **25**(2), 208–218 (2022). 34088994 10.1038/s41391-021-00398-1

[22] Melton, L. J. III et al. Fracture risk in men with prostate cancer: A population-based study. *J. Bone Min. Res.* **26**(8), 1808–1815 (2011). 10.1002/jbmr.405 PMC3321611 21520274

[23] Kanis, J. A., Johansson, H., Oden, A. & McCloskey, E. V. Assessment of fracture risk. *Eur. J. Radiol.* **71**(3), 392–397 (2009). 19716672 10.1016/j.ejrad.2008.04.061

[24] Welfare MoHa. Patients with Catastrophic Illnesses or Rare Diseases (2022).

[25] Welfare MoHa. *The Items of Catastrophic Illness under National Health Insurance and Their Certificate Validity Period* (Ministry of Health and Welfare, 2023).

[26] Morote, J. et al. Prevalence of osteoporosis during long-term androgen deprivation therapy in patients with prostate cancer. *Urology*. **69**(3), 500–504 (2007). 17382153 10.1016/j.urology.2006.11.002

[27] Cianferotti, L. et al. The prevention of fragility fractures in patients with non-metastatic prostate cancer: A position statement by the international osteoporosis foundation. *Oncotarget*. **8**(43), 75646–75663 (2017). 29088899 10.18632/oncotarget.17980 PMC5650454

[28] Rizzoli, R. et al. Cancer-associated bone disease. *Osteoporos. Int.* **24**(12), 2929–2953 (2013). 24146095 10.1007/s00198-013-2530-3 PMC5104551

[29] Gralow, J. R. et al. NCCN task force report: Bone health in cancer care. *J. Natl. Compr. Canc Netw.* **11**(Suppl 3), S1–50 (2013). quiz S51. 23997241 10.6004/jnccn.2013.0215

[30] Suarez-Almazor, M. E. et al. Association of bone mineral density testing with risk of major osteoporotic fractures among older men receiving androgen deprivation therapy to treat localized or regional prostate cancer. *JAMA Netw. Open.* **5**(4), e225432 (2022). 35363269 10.1001/jamanetworkopen.2022.5432 PMC8976238

[31] Hu, J., Aprikian, A. G., Vanhuyse, M. & Dragomir, A. Contemporary population-based analysis of bone mineral density testing in men initiating androgen deprivation therapy for prostate cancer. *J. Natl. Compr. Canc Netw.* **18**(10), 1374–1381 (2020). 33022649 10.6004/jnccn.2020.7576

[32] Hussain, M. et al. Enzalutamide in men with nonmetastatic, castration-resistant prostate cancer. *N. Engl. J. Med.* **378**(26), 2465–2474 (2018). 29949494 10.1056/NEJMoa1800536 PMC8288034

[33] Smith, M. R. et al. Apalutamide and overall survival in prostate Cancer. *Eur. Urol.* **79**(1), 150–158 (2021). 32907777 10.1016/j.eururo.2020.08.011

[34] Fizazi, K. et al. Darolutamide in nonmetastatic, castration-resistant prostate cancer. *N. Engl. J. Med.* **380**(13), 1235–1246 (2019). 30763142 10.1056/NEJMoa1815671

[35] Fizazi, K. et al. Nonmetastatic, castration-resistant prostate cancer and survival with darolutamide. *N. Engl. J. Med.* **383**(11), 1040–1049 (2020). 32905676 10.1056/NEJMoa2001342

[36] Clegg, N. J. et al. ARN-509: A novel antiandrogen for prostate cancer treatment. *Cancer Res.* **72**(6), 1494–1503 (2012). 22266222 10.1158/0008-5472.CAN-11-3948 PMC3306502

[37] Moilanen, A. M. et al. Discovery of ODM-201, a new-generation androgen receptor inhibitor targeting resistance mechanisms to androgen signaling-directed prostate cancer therapies. *Sci. Rep.* **5**, 12007 (2015). 26137992 10.1038/srep12007 PMC4490394

[38] Merriman, J. D., Von Ah, D., Miaskowski, C. & Aouizerat, B. E. Proposed mechanisms for cancer- and treatment-related cognitive changes. *Semin Oncol. Nurs.* **29**(4), 260–269 (2013). 24183157 10.1016/j.soncn.2013.08.006 PMC3817493

[39] Williams, S. C. R. et al. Comparison of cerebral blood flow in regions relevant to cognition after enzalutamide, darolutamide, and placebo in healthy volunteers: A randomized crossover trial. *Target. Oncol.* **18**(3), 403–413 (2023). 37103658 10.1007/s11523-023-00959-5 PMC10191908

