# Supplementary material for: The impact of novel hormonal agents on fracture risk in prostate cancer patients: a nationwide population-based cohort study
Source: Sci Rep. 2024 Nov 4;14:26696. doi: 10.1038/s41598-024-73598-z (PMC11535545; doi:10.1038/s41598-024-73598-z)
Supplement: Supplementary file 1 — Supplementary Material 1 [file 41598_2024_73598_MOESM1_ESM.docx]

**Supplement Figure 1.** Flowchart of Enrollment and Exclusion Criteria for Newly Diagnosed Prostate Cancer Patients

**Figure Legend**

This flowchart describing the enrollment and exclusion process for newly diagnosed prostate cancer patients. Patients with evidenced metastatic disease were excluded. Additionally, patients with any fractures 12 months prior to or 6 months after the initiation of ADT were excluded to clarify the osteoporotic effects of ADT and minimize underlying fragility issues. The remaining patients were divided into the ADT alone group and the NHA combination group.

Newly diagnosed patients with prostate cancer (ICD9-CM code: 185/ ICD10-CM code: C61) with registry of catastrophic illness between 2000 and 2019

n= 81474

Excluded:

- Secondary malignant neoplasm of bone: ICD9: 198.5/ ICD10: C79.51 (n= 165)
- Diagnosed with M1b based on Taiwan Cancer Registry database (n= 7476)

Androgen deprivation therapy (ADT) alone

n= 25166

Novel hormonal agents combination therapy

n= 783

Patients treated with androgen deprivation therapy (ADT) between 2000/1/1 and 2018/6/30
n= 31054

Excluded:

- Any bone fracture that takes place within 12 months prior to or 6 month after initiating ADT(n=2628)
- Sequential use of bisphosphonate and denosumab(n= 825)
- Death within 6 month after initiating ADT (n=1652)

Patients with prostate cancer
n= 73833
